# Comparative Assessment of Preoperative versus Postoperative Dexamethasone on Postoperative Complications following Lower Third Molar Surgical Extraction

**DOI:** 10.1155/2017/1350375

**Published:** 2017-04-10

**Authors:** Hashem M. Al-Shamiri, Maha Shawky, Nermin Hassanein

**Affiliations:** ^1^Department of Oral and Maxillofacial Surgery Sciences, Alfarabi Colleges, Riyadh, Saudi Arabia; ^2^Oral and Maxillofacial Surgery Department, Faculty of Dentistry, King Abdulaziz University, Jeddah, Saudi Arabia; ^3^Faculty of Oral and Dental Medicine, Cairo University, Giza, Egypt; ^4^Oral and Maxillofacial Surgery Department, Faculty of Dentistry, Faculty of Oral and Dental Medicine, Cairo University, Giza, Egypt

## Abstract

*Aim*. To evaluate the effect of preoperative versus postoperative administration of oral Dexamethasone on postoperative complications including pain, edema, and trismus following lower third molar surgery.* Methods*. 24 patients were divided into two equal groups receiving 8 mg Dexamethasone orally, one group one hour preoperatively and the other group immediately after surgery. Pain was measured using VAS, edema was measured using a graduated tape between 4 fixed points in the face, and the mouth opening was measured using a graduated sliding caliper.* Results*. In this study pain and trismus records were similar and statistically nonsignificant in both groups. The results had proven that preoperative administration was superior when compared to postoperative administration regarding edema (0.002).* Conclusions*. Preoperative oral administration of 8 mg Dexamethasone was superior to the postoperative administration of the same dose concerning edema after lower third molar surgery.

## 1. Introduction

The surgical extraction of lower third molars is the most frequent intervention in oral surgery [1]. This procedure is often associated with significant postsurgical sequelae that may have both biological and social impact. Besides severe complications such as dysaesthesia, severe infection, fracture, and dry socket, patients frequently complain of pain, swelling, and limitation in mouth opening (Trismus) throughout the postoperative course due to the inflammatory response following the surgical injury [2].

Surgical removal of lower third molar can vary in difficulty and in the degree of trauma caused to the surrounding tissue. The greater amount of tissue injury leads to an increased amount of inflammation in the peri-surgical area. Swelling may be particularly significant when the surgery is prolonged and when large amounts of bone, gingiva, and oral mucosa are manipulated. Careful surgical technique is effective in limiting tissue damage and swelling; therefore, attention should be taken to avoid prolonged periods of tissue elevation and retraction [3].

Postoperative pain following surgical removal of impacted mandibular third molar is a localized inflammatory pain of varying intensity. The removal of impacted mandibular third molar with the resultant tissue and cellular destruction brings about the release and production of several biochemical mediators; these mediators involved in the pain process are histamine, bradykinin, and prostaglandins which are a group of biologically active fatty acids [4]. Pain after surgery of impacted lower third molar is reduced by using analgesics and long-acting local anesthetics. Another method of reducing postoperative pain is the proper surgical technique with careful flap reflection and using irrigation for cooling [5].

Postsurgical edema is a normal physiological reaction to insult and injury. When body tissues are injured, regardless of the cause, the normal physiologic response is inflammation, leading to edema. The amount of the postoperative edema varies according to local factors as position of the impacted teeth, method of bone removal, haemostasis, oversuturing of the wound, or rough tissue handling and systemic factors as age, bleeding tendency, nutrition, use of drugs, or presence of diabetes [2]. The use of corticosteroids to limit postoperative edema has been advocated due to their inhibitory action on signal transduction through IL-2 receptor [6].

To control postoperative inflammation and symptoms associated, it is necessary to provide an adequate anti-inflammatory therapy [2]. The use of corticosteroids can decrease the severity of postoperative sequelae in many patients and therefore decrease morbidity after oral surgery [3]. For more than 30 years, glucocorticosteroids have been used in an attempt to minimize or prevent postoperative sequelae after surgical removal of impacted third molars and several studies [7–10] have been published in the literature on this subject.

Some studies [7, 11–13] compared the use of Dexamethasone in different formulations one hour before surgery in patients undergoing extraction of third molars and observed the postoperative edema, trismus, and pain. From this study, it was concluded that there was no significant difference between the two formulations of Dexamethasone in surgery of third molars in relation to its use as a preoperative medication to reduce swelling, trismus, and pain.

The aim of this study was to evaluate the effect of preoperative versus postoperative administration of oral Dexamethasone on postoperative discomfort including pain, edema, and trismus following lower third molar surgery in order to allow a better welfare of the patient and to return the patient to the normal activity following the surgery.

## 2. Materials and Methods

The study was conducted on 24 healthy patients requiring surgical removal of the mandibular impacted third molar under local anesthesia among patients attending the out-patient clinic, Oral and Maxillofacial Surgery Department Cairo University. Of total, 12 were males and 12 were females.

The inclusion criteria of the selected patient were freedom of any systemic diseases that could interfere with wound healing or surgical operation, freedom of any recent anti-inflammatory drug intake or being under long term treatment with medicaments that will obscure the assessment of the inflammatory response as NSAIDs, steroids, or antihistamines, and freedom from allergy to the drugs used in the study.

All patients were subjected to clinical and radiographic examination with single examiner (periapical and panorex view) and the study protocol was explained to the patients in detail. Subjects who met the criteria of the study were informed of the operative procedure and postoperative instructions after which a written consent was obtained before surgery.

After designating a specific number for each case, they were randomly distributed into two groups using a research randomization program in the computer (group A and group B) of 12 patients in each. Patients in group A were given 8 mg (2 tablets of 4 mg) Dexamethasone (Dexamethasone Tablets USP, 4 mg, Roxane Laboratories, Inc., Columbus, OH, USA) orally 1 hour before the surgery, while for group B, patients were given 8 mg (2 tablets of 4 mg) orally immediately after completion of the surgery.

**Figure 1:**
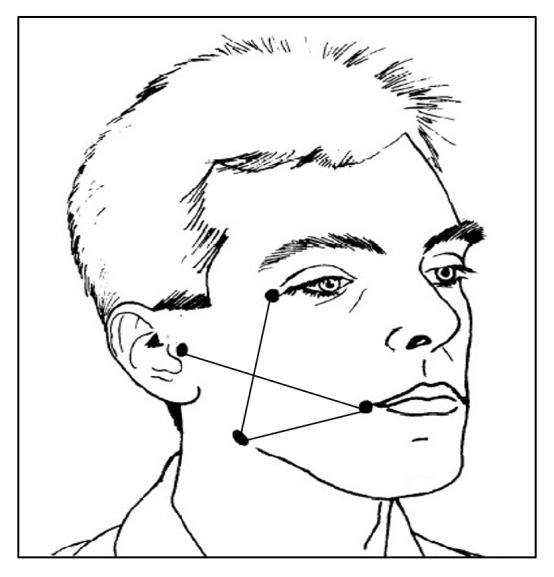
Diagram of the four fixed anatomical references, namely, outer canthus of eye, tragus of the ear, gonion, and corner of the mouth.

Preoperative facial measurements were taken in three planes using a tape measuring method; the first plane was from tragus of the ear to the corner of the mouth, the second plane form the gonion to corner of the mouth, and the third plane from the outer canthus of the eye to the gonion (Figure 1). The measurements were taken with the patient seated in an upright position with the teeth lightly in occlusion. The graduated tape used to measure the lines was neither tensed nor relaxed and would follow exactly the facial contour with gentle skin touch. The preoperative sum of the three measurements was considered as the baseline for that side. Then the mean was recorded for these three records. These facial measurements were repeated at the 2nd, 5th, and 7th days postoperatively. Also the maximum interincisal distance was recorded both preoperatively and at the 2nd, 5th, and 7th days postoperatively using a graduated sliding caliper for measuring the interincisal distance between the mesioincisal angle of upper central incisor and that of lower incisor at maximum mouth opening taking them as a baseline for measurements. Trismus was recorded as the difference in interincisal distance at maximum opening before and after the operation. Pain measurements were recorded using a visual analogue scale (VAS), which consists of plan horizontal 10 mm long line starting from “No Pain” at one end (0) point and the “Worst Pain” at the other end at (10) point, and patients were asked to mark each scale according to their pain at a given time at night the day of the surgery and two days postoperatively both in the morning and at night.

The surgical procedures were carried out by the same operator under local anesthesia (Mepevicaine HCl 2% with Levonordefrin 1 : 200,000). A three-sided mucoperiosteal flap was reflected, buccal bone guttering was performed under a copious irrigation with sterile isotonic saline, and tooth division was done when indicated using surgical burs of suitable size. The tooth was delivered from the socket and the sharp bone was smoothed and the socket was irrigated with isotonic saline; then the flap was repositioned and sutured using 3/0 black silk, which was removed after 7 days of surgery. The duration of the surgery and osteotomy was recorded as the period between the incision and the last suture.

All patients received the routine postoperative instructions and standard antibiotic therapy (Clindamycin 300 mg tid. for 5 days) and analgesic (Ibuprofen 400 mg tid. for 3 days) and instructed to request additional analgesics tabs in the event of aggravated pain episodes. Follow-up was carried out on the 2nd, 5th, and 7th days postoperatively.

### 2.1. Statistical Analysis

Data were analyzed using PASW Statistics 18.0 (Predictive Analytics Software) for windows (SPSS: An IBM Company, Chicago, IL, USA). Numerical data were presented as mean and standard deviation values, where Student's and paired *t*-tests were used for parametric numerical data, but Mann–Whitney* U* test and Wilcoxon signed-rank test were used for nonparametric numerical data.

Qualitative data were presented as frequencies and percentages; Chi-square (*χ*^2^) test was used between the two groups. The significance level was set at *p* ≤ 0.05.

## 3. Results

This study was conducted on 24 patients (mean age 26.7 years), including 12 males (50%) and 12 females (50%) requiring surgical removal of the impacted lower third molar tooth. Wound healing in all patients was uneventful; no bleeding, infection, or delayed wound healing was observed. No side effects as discomfort, nausea, vomiting, headache, epigastric discomfort, or gastrointestinal irritations were reported by the patients in both groups concerning the drug used in the study and all patients were able to resume their normal activities on the second day after surgery.

**Figure 2:**
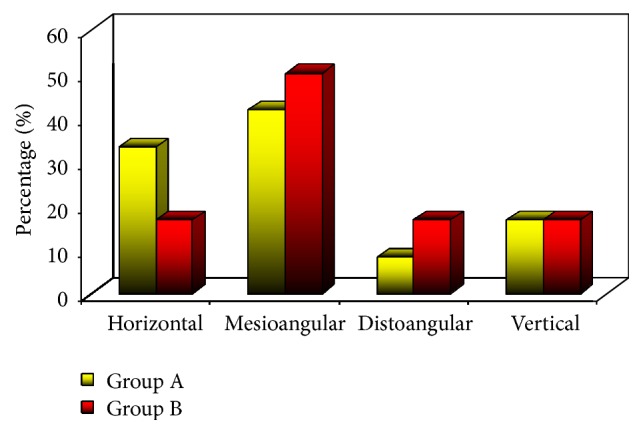
Directions of impaction in the two groups.

**Figure 3:**
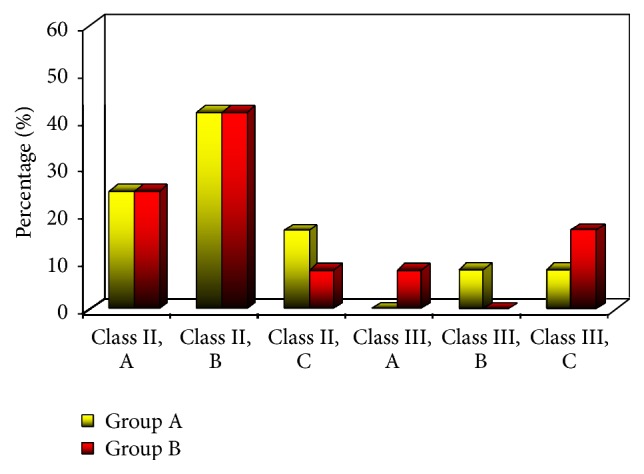
Types of impaction in the two groups.

The radiographic analysis of the angulation (*p value*, 0.779) and depth (*p value*, 0.751) of the impacted teeth according to Winter's classification showed no statistically significant difference between types of impaction in the two groups (Figures 2 and 3).

**Figure 4:**
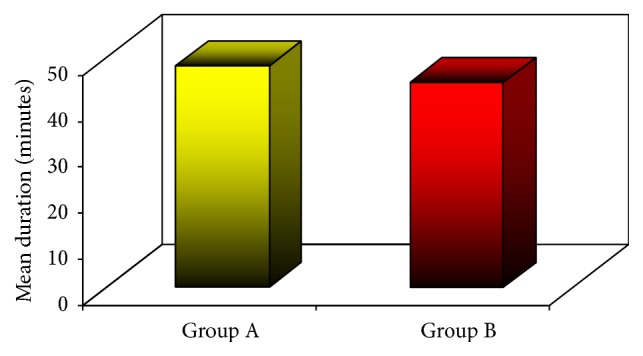
Mean duration of operation in the two groups.

The mean of the duration of operation in group (A) was 48.3 minutes, while the values were 44.6 minutes in group B, with no statistically significant difference (*p value,* 0.292) (Figure 4).

**Figure 5:**
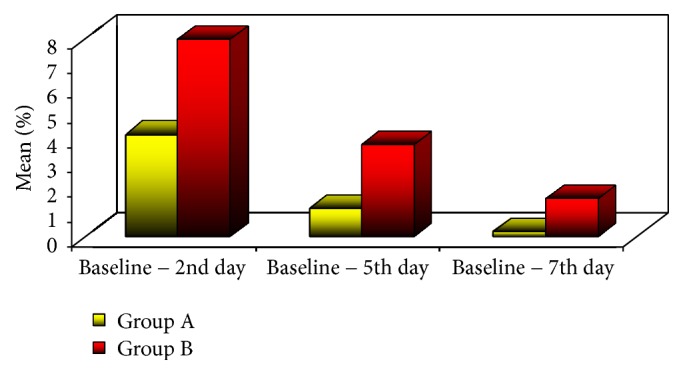
Mean% change in edema measurement in the two groups.

For edema, through all periods, group B showed statistically significant higher mean% increase in edema measurement than group A (*p value,* 0.003) (Table 1) (Figure 5).

**Figure 6:**
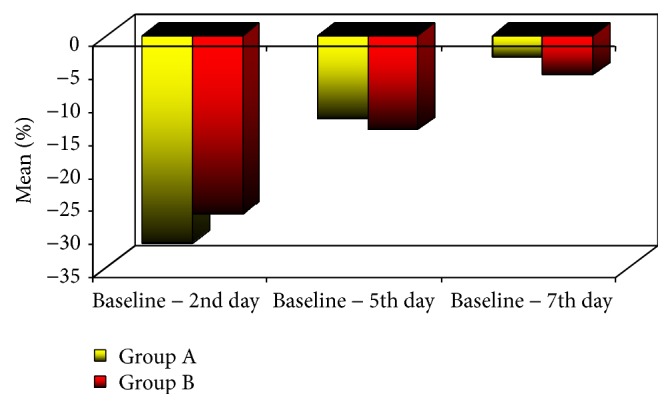
Mean% change in MIO of the two groups.

For trismus, only after 7 days, group B showed statistically significant higher mean% reduction in maximum interincisal opening value than group A (*p value,* 0.021) (Table 1) (Figure 6).

**Figure 7:**
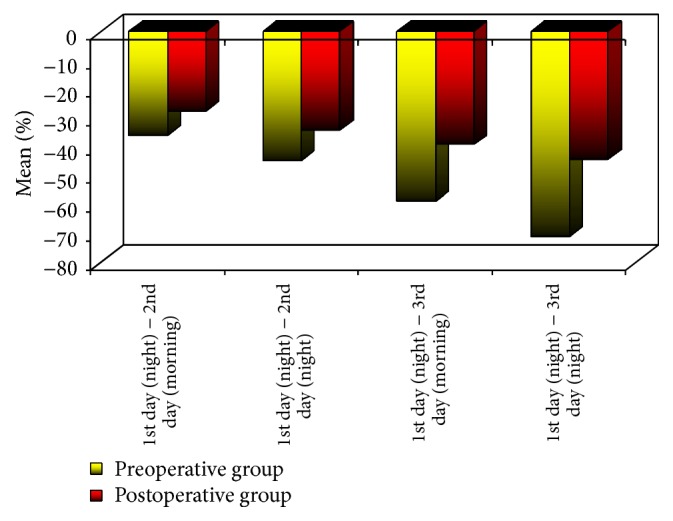
Mean% decrease in VAS of the two groups.

With regard to pain, at the 3rd day in the morning and night, group A showed statistically significant higher mean% decrease in VAS than group B (*p value* on morning, 0.008;* p value* on night, 0.009) (Table 2) (Figure 7).

## 4. Discussion

Prevention and management of postoperative consequences following lower third molar surgery are an essential part of the clinical practice; thus, many attempts have been made to reduce these sequelae by using the anti-inflammatory drugs.

The anti-inflammatory effects of glucocorticoids are well-documented; however, how exactly the steroid influences inflammation is not completely understood and is a continuing area of investigation. The primary mechanisms are thought to involve suppression of leukocyte and macrophage accumulation at the site of the inflammation and prevention of prostaglandins formation [14].

Prostaglandins are inhibited by the disruption of the arachidonic acid cascade. Lipocortin, an endogenous protein produced by steroids, blocks the activity of phospholipase A2, thus influencing the release of arachidonic acid from cell membranes and the synthesis of prostaglandins, leukotrienes, and thromboxane [15].

All routes of administration of corticosteroids have given significant improvement in pain and swelling unless otherwise when the Dexamethasone is contraindicated. Use of the corticosteroid, Dexamethasone, given by local [16], oral [11, 17, 18], intramuscular [19], intravenous, or submucosal [11, 17, 20, 21] routes, either preoperatively, perioperatively, or postoperatively appears to be effective in the prevention of postoperative edema. Long-acting steroids give better results than short-acting one and submucosal administration of steroids produces similar effects to intravenous and intramuscular routes [22].

In this study, Dexamethasone was chosen because of its higher potency, lower sodium-retaining ability, and longer half-life [23]. For the dose we choose the 8 mg was the least amount with best benefits that can be achieved, as the normal daily output of cortisol is 15–25 mg/day, but up to 300 mg of cortisol can be released in a time of crisis, and the 8 mg Dexamethasone is nearly equivalent to this amount of released cortisol [24]. The method used was simple, applicable, and easily accepted by the patients.

The age, gender, type of impaction, and duration of operation might be significant risk indicators for postoperative morbidity, as they are commonly reported to be significant to the occurrence of complications. In this study there was no statistically significant difference between respective mean values in the two groups. So these parameters in this study did not have a significant effect in the comparison between the two groups.

Postsurgical facial edema is difficult to quantify accurately because it involves 3 dimensions of measurement with an irregular, convex surface and can manifest itself internally as well as externally [25].

Edema was at a maximum on the second postoperative day in the two groups. This swelling was reduced gradually but lasted for 2–5 days; this result is in agreement with further studies [5, 7, 8]. The edema subsided almost completely after one week in both groups, particularly in group A. This early resolution of edema in group A could be attributed to the early action of Dexamethasone, as it reaches its peak action in the serum with oral route within 1-2 hours, and its biological half-life reaches 36–54 hours [26].

Trismus measured in this study as a decrease in maximal interincisal opening is a significant postoperative sequela that is attributed to the edema, swelling, and pain associated with the surgical trauma. Restriction of mouth opening can be caused by the splinting action of the investing muscles in an attempt to reduce discomfort upon jaw movement after surgery or due to the inflammation widespread involving the muscles of mastication with edema preventing its flexibility [27]. The time course for trismus and concurrent limitation in oral function described in the current study are in agreement with findings that indicated that trismus reaches a maximum on Day 1 or Day 2 postoperatively and generally resolve by Day 7 [11, 21]. After 7 days, group B showed statistically significantly higher mean% reduction in maximum interincisal opening value than group A (45.3 ± 1.5 in A, 44.2 ± 6.1 in B,* p value*, 0.527). This increase in the maximum interincisal opening in group A in the 7th day in comparison to group B may be attributed to the fact that the swelling or edema in group A is almost absent, thus allowing more free movement and increased opening of the jaws. At one week, the maximum interincisal opening was not different from preoperative measurement in the two groups, especially in group A. This finding is in agreement with the findings of previous reports [28].

Acute postoperative pain following third molar surgery is predominantly a consequence of inflammation caused by tissue injury [29]. Its course depends on the degree of surgical trauma suffered, the need for bone tissue removal, and the extension of periosteum displacement [8].

This study showed a statistically significant decrease in mean VAS by time in group A (−59.1% in the morning and −71.4% in the night) compared to group B (−39.3% in the morning and −44.5% in the night) at the 3rd day after surgery. This difference may be attributed to the fact that, in group A, the Dexamethasone acts early in the inflammatory area, thus decreasing the production of inflammatory mediators at the area of surgery, and this leads to a more gradual reduction of the swelling and edema in that area, which brings good control of early postoperative pain and more comfort for the patients who gave low records in VAS score. But generally the evaluation of pain through the 3 postoperative days was regressive for the two groups, and this coincides with some studies [7, 8] who stated that in the 7th day of the surgery pain approaches zero.

Inflammatory complications after third molar surgery still remain an important factor in quality of life of patients at the early postoperative periods [30]. Oral surgeons should be aware of the different modalities of alleviation of these complications to make postoperative recovery more comfortable for patient.

## 5. Conclusion

Based on the findings of the present study, the oral administration of 8 mg Dexamethasone either preoperatively or postoperatively reduces the postoperative complications as pain, edema, and trismus associated with lower third molar surgeries. Although in this study pain and trismus records were comparable and statistically nonsignificant in both groups, there was a significant reduction in pain records and trismus in the preoperative group at the third and seventh days, respectively, compared to the postoperative one. Furthermore, the results had proven that preoperative oral administration of 8 mg Dexamethasone was superior to the postoperative administration of the same dose in reducing edema following lower third molar surgery.

## Figures and Tables

**Table 1:** Mean, SD, and difference by group of maximum mouth opening and edema.

	Evaluation/difference	Groups		*p* value
		Group A	Group B	
		Mean ± SD	Mean ± SD	
Maximum mouth opening	Baseline	46.9 ± 1.4	46.9 ± 6.2	1.000
	2nd day	32.2 ± 5.7	34.6 ± 8.2	0.412
	5th day	41 ± 2.9	40.3 ± 6.7	0.756
	7th day	45.3 ± 1.5	44.2 ± 6.1	0.527
	*Difference*			
	Baseline − 2nd day	−31.4 ± 12.6	−26.9 ± 10.5	0.371
	Baseline − 5th day	−12.5 ± 6.9	−14.2 ± 5.8	0.563
	Baseline − 7th day	−13.3 ± 4.3	−5.9 ± 1.9	0.021^*∗*^

Edema	Baseline	9.84 ± 0.5	9.77 ± 0.9	0.815
	2nd day	10.24 ± 0.3	10.53 ± 0.9	0.307
	5th day	9.95 ± 0.4	10.12 ± 0.9	0.548
	7th day	9.86 ± 0.4	9.92 ± 0.9	0.847
	*Difference*			
	Baseline − 2nd day	4.1 ± 2.2	7.9 ± 3	0.003^*∗*^
	Baseline − 5th day	1.1 ± 1	3.7 ± 2.2	0.003^*∗*^
	Baseline − 7th day	0.2 ± 0.5	1.5 ± 1.8	0.038^*∗*^

^*∗*^Significant at *p* ≤ 0.05.

**Table 2:** Mean, SD, and difference by group of visual analogue scale (VAS).

Variable	Evaluation/difference	Groups		*p* value
		Group A	Group B	
		Mean ± SD	Mean ± SD	
VAS scores	1st day (night)	6.2 ± 2.5	5.8 ± 2.4	0.556
	2nd day (morning)	4.2 ± 2.5	3.8 ± 1.9	0.815
	2nd day (night)	3.7 ± 2.1	3.4 ± 1.4	0.976
	3rd day (morning)	2.6 ± 1.5	3.2 ± 1.5	0.321
	3rd day (night)	1.8 ± 1.6	1.8 ± 0.7	0.462
	*Difference*			
	1st day (night) − 2nd day (morning)	−36 ± 20.1	−27.5 ± 26.2	0.458
	1st day (night) − 2nd day (night)	−45 ± 16.4	−34.3 ± 19.6	0.152
	1st day (night) − 3rd day (morning)	−59.1 ± 12.7	−39.3 ± 20.5	0.008^*∗*^
	1st day (night) − 3rd day (night)	−71.4 ± 15.9	−44.5 ± 36.2	0.009^*∗*^

^*∗*^Significant at *p* ≤ 0.05.
